# Medical Society of Logan, Mason, and Menard Counties, Ills.

**Published:** 1850-07

**Authors:** 


### ARTICLE V.

### MEDICAL SOCIETY OF LOGAN, MASON, AND MENARD COUNTIES, ILLINOIS.

We have received a copy of the proceedings of the Convention that organized a Society by the above name, held on he 11th of February last.

Dr. James Smick was elected President, Dr. James P. Walker, Vice-President, Dr. Chamberlain, Secretary, and Dr. Patterson, Treasurer.

The first annual meeting was to have been held on the second Wednesday of May last, at Middletown. Although from inadvertance and irregularity of the mail, this notice is late in making its appearance, we wish our friends abundant success. E.

